# Geographical Disparities in HIV Seroprevalence Among Men Who Have Sex with Men and People Who Inject Drugs in Nigeria: Exploratory Spatial Data Analysis

**DOI:** 10.2196/19587

**Published:** 2021-05-24

**Authors:** Amobi Onovo, Abiye Kalaiwo, Moses Katbi, Otse Ogorry, Antoine Jaquet, Olivia Keiser

**Affiliations:** 1 Institute of Global Health University of Geneva Geneva Switzerland; 2 U S Agency for International Development Abuja Nigeria; 3 The President's Emergency Plan For AIDS Relief Coordination Office Abuja Nigeria; 4 Institut de Santé Publique d'épidémiologie et de développement University of Bordeaux Bordeaux France

**Keywords:** key population, MSM, PWID, HIV seroprevalence, HIV testing modality, hotspots, geospatial, Getis-Ord-Gi*, IBBSS, Nigeria

## Abstract

**Background:**

The assessment of geographical heterogeneity of HIV among men who have sex with men (MSM) and people who inject drugs (PWID) can usefully inform targeted HIV prevention and care strategies.

**Objective:**

We aimed to measure HIV seroprevalence and identify hotspots of HIV infection among MSM and PWID in Nigeria.

**Methods:**

We included all MSM and PWID accessing HIV testing services across 7 prioritized states (Lagos, Nasarawa, Akwa Ibom, Cross Rivers, Rivers, Benue, and the Federal Capital Territory) in 3 geographic regions (North Central, South South, and South West) between October 1, 2016, and September 30, 2017. We extracted data from national testing registers, georeferenced all HIV test results aggregated at the local government area level, and calculated HIV seroprevalence. We calculated and compared HIV seroprevalence from our study to the 2014 integrated biological and behavioural surveillance survey and used global spatial autocorrelation and hotspot analysis to highlight patterns of HIV infection and identify areas of significant clustering of HIV cases.

**Results:**

MSM and PWID had HIV seroprevalence rates of 12.14% (3209/26,423) and 11.88% (1126/9474), respectively. Global spatial autocorrelation Moran I statistics revealed a clustered distribution of HIV infection among MSM and PWID with a <5% and <1% likelihood that this clustered pattern could be due to chance, respectively. Significant clusters of HIV infection (Getis-Ord-Gi* statistics) confined to the North Central and South South regions were identified among MSM and PWID. Compared to the 2014 integrated biological and behavioural surveillance survey, our results suggest an increased HIV seroprevalence among PWID and a substantial decrease among MSM.

**Conclusions:**

This study identified geographical areas to prioritize for control of HIV infection among MSM and PWID, thus demonstrating that geographical information system technology is a useful tool to inform public health planning for interventions targeting epidemic control of HIV infection.

## Introduction

Geographic variation in HIV seroprevalence has been demonstrated in many sub-Saharan African countries [1-7]. According to the Nigeria AIDS Indicator and Impact Survey (NAIIS) 2018, HIV seroprevalence in Nigeria among adults aged 15-64 years was 1.5% (1.9% among females and 1.1% among males), ranging from 0.6% in the North West to 3.1% in the South South zone [8]. The spatial distribution of HIV infection in key populations (KPs), including in men who have sex with men (MSM) and people who inject drugs (PWID), is much less understood. Nigeria has a mixed epidemic, meaning that while HIV prevalence among the general population is high, certain groups still carry a far more significant HIV burden than the rest of the population. Men who have sex with men are the only group in Nigeria where HIV prevalence is still rising. In 2017, this group's prevalence stood at 23%, significantly more than the next highest prevalence group—sex workers— at 14.4% [9]. Of all new HIV infections in the country, 10% occur among MSM [10]. HIV prevalence among people who inject drugs (sometimes referred to as PWID) in Nigeria was 3.4% in 2017 [11]. Women who inject drugs are particularly affected, with a prevalence of 13.9% compared to 2.6% among men [9]. Female sex workers who inject drugs face the highest HIV prevalence at around 43% [9]. According to the 2010 integrated biological and behavioral surveillance survey (IBBSS) in Nigeria, female injecting drug users had a 7-fold higher HIV prevalence than male injecting drug users [12].

In, sub-Saharan Africa, MSM are at increased risk for HIV infection compared to men in the general population [13]. The HIV epidemic in West Africa is mainly heterosexually driven, but recent data suggest that anal sex plays a significant independent role in HIV infection [14]. In the 2015 the Joint United Nations Programme on HIV/AIDS (UNAIDS) report, studies in sub-Saharan Africa indicated prevalence rates of HIV infection ranging from 6% to 37% among MSM [15]. According to the progress reports on the global AIDS response, the highest prevalence rates of HIV infection among MSM were as follows: 19% in Central and Western Africa, 15% in Southern and Eastern Africa, 12% in Latin America, 11% in the Asia-Pacific region, and 8% in Central and Western Europe and North America [16]. A recent study conducted among MSM across India reported a relatively high HIV prevalence of 7% [17]. These reports indicate that a substantial number of infections occur among MSM, many of whom also have sex with women [18]. Other studies have confirmed that MSM who engage in transactional sex have an increased HIV prevalence compared to MSM who do not engage in transactional sex [17,19]. The variability in HIV prevalence may be attributed to individual, social, and structural level factors. MSM who engage in transactional sex may not be responsive to HIV prevention services designed explicitly for gay-identified MSM. These men are affected by similar multilevel factors, such as stigma and discrimination, inability to negotiate condom use, substance use and misuse, and psychological distress [20]. MSM across many countries in sub-Saharan Africa face stigma and discrimination [21]. Stahlman et al's [22] study revealed that stigmatization as gay and fear of being HIV positive present barriers to accessing available voluntary HIV testing, counseling, and treatment services. In Nigeria, the Same-Sex Marriage Prohibition law was passed by the Senate in 2011 and was implemented in January 2014. The new law criminalized same-sex practices, prohibiting participation in organizations, service provision, or meetings that support gay people, and instituting punishments for attempts to enter civil unions or publicly show same-sex relationships [23].

About one-third of global HIV infections outside sub-Saharan Africa are related to PWID, who account for a growing proportion of persons living with HIV. It is estimated that PWID are 22 times more likely to acquire HIV than those among the rest of the population [24]. This risk arises from sharing needles and injection equipment but is reinforced through criminalization, marginalization, and poverty. HIV prevalence among PWID in Nigeria was 3.4% in 2017 [10]. Women who inject drugs are particularly affected, with a prevalence of 13.9% compared to 2.6% among men [10]. Seroprevalence ranges from 3.4% in Nigeria to 8.5% in Sierra Leone [10]. However, Nigeria has the highest number of PWID in the West African region, estimated at 45,000 in 2017 [10]. A recent study in 2015 among PWID showed that the estimated HIV prevalence was 18.1% [25]. HIV seroprevalence among women who inject drugs is much higher than among men who inject drugs. For example, in Senegal, the HIV seroprevalence among women and men who inject drugs is 28% and 7%, respectively [26].

Previous studies, have focused on developing statistical models to predict the seroprevalence of HIV among KPs in sub-Saharan Africa [27,28]. These models included multiple risk factors (eg, circumcision, distance to road, and condom usage). They have shown that there can be considerable small-scale heterogeneity in HIV seroprevalence and risk behaviors. However, none of these models have tested whether there is a spatial pattern of HIV infection among KPs, and the size of the studies has been limited. In our study, we examined the spatial pattern of HIV prevalence among MSM and PWID in Nigeria. There is currently great interest amongt public health planners concerning whether this geographic variation of HIV could be used to improve the effectiveness of targeted HIV prevention interventions by focusing on areas of highest need [29-31]. The study is divided roughly into 3 phases, each of which covers a particular study objective. The first phase used an HIV testing program data to estimate the seroprevalence of HIV between MSM and PWID, the second phase used geographic information system techniques to assess the infection pattern and classify HIV infection hot spots, and the third phase compared the outcomes of HIV seroprevalence from program data and the last IBBSS 2014 [32] carried out in Nigeria by the Federal Ministry of Health.

Therefore, we aimed to quantify the geographic variation of HIV seroprevalence among MSM and PWID by age and sex through using georeferenced HIV testing services data aggregated at the level of local government areas (LGAs). We planned to detect hot spots and compare HIV seroprevalence from our study to that of the 2014 IBBSS. We defined HIV seroprevalence as the overall occurrence of HIV within a defined population at a particular time, as measured by blood or serologic tests. The IBBSS was scheduled to occur every 5 years, with the last one taking place in 2014. The survey results served as the primary data source for the national response to AIDS among KPs in Nigeria. To track changes in HIV infection among MSM and PWID in the selected study and survey states, we compared HIV seroprevalence from our study with HIV prevalence from the 2014 IBBSS.

## Methods

### Study Setting and Data Source

Nigeria is organized into 36 federating states and the Federal Capital Territory, which hosts the national government. The states are further subdivided into 774 LGAs. The study was conducted in 93 LGAs spread across 7 prioritized states (Akwa Ibom, Rivers, Cross Rivers, Benue, Nasarawa, Lagos, and the Federal Capital Territory) in the 3 geographic regions of North Central, South South, and the South West. Under the national KP program, these states were prioritized for prevention and comprehensive HIV/AIDS treatment interventions due to their high population density and the high number of people living with HIV. The national KP program consists of an integrated HIV prevention and treatment program that identifies HIV-positive KPs in the community and links them to care and treatment at the LGA level. The primary source of data for this study was the performance HIV testing services data (disaggregated by fine age bands and sex) obtained from a Nigerian KP program, the Integrated Most-At-Risk Populations HIV/AIDS Prevention Program [33].

### HIV Testing Algorithm

HIV testing delivery services to KPs in the target communities adopted a 2-step HIV rapid testing serial algorithm testing strategy. Fourth generation rapid test kits were used for HIV testing (Alere Determine HIV-1/2 Ag/Ab Combo, Alere Scarborough, Inc). A nonreactive HIV test result after the first test was considered HIV negative. A reactive result was tested with a second test. When the result was reactive again, it was considered HIV positive, and when it was nonreactive, it was considered HIV negative. All HIV test results were documented using the national HIV testing and counseling register. Based on program design and data reporting requirements, the HIV test results of each KP (MSM and PWID) were aggregated up by the program level performance indicator, “Number of individuals who received HIV Testing Services and received their test result,” and linked to the location or LGA where the KP was tested.

### HIV Testing Modalities for KPs

The national HIV/AIDS program provides targeted HIV testing services to KP and their high-risk contacts. The following services are provided: index partner or index testing (also referred to as partner testing or partner notification) in which exposed contacts of an HIV-positive index case are offered HIV testing; KP testing in mobile or temporary testing locations, such as community centers, schools, workplaces, hotels, clubs, tents, and vans; and voluntary counseling and testing, which includes testing in voluntary counseling and testing centers outside of a health facility (ie, “one-stop shops”). The one-stop shop model for KP establishes safe spaces in the communities for HIV prevention and treatment interventions. It integrates differentiated strategies that optimize efficiency along the 90-90-90 cascade [34]. HIV test results are aggregated by the KP group and the LGA where the test is conducted. To reduce double-counting of individuals and to account for retesters in a reporting period, tracking systems, such as “unique identifiers”, are established and used to monitor the frequency of contact or outreach of the KPs over time. A unique ID is generated and assigned to a KP group before HIV testing commences, and the results collected are entered into an electronic database. Data validation is conducted every quarter by running a query of all individual-level KP data on HIV testing using IDs to determine first-time testers and repeat testers. All duplicated KP data within the reporting period are deduplicated before data transmission. This is accomplished by community-level data review and reconciliation exercises between testing counselors and monitoring and evaluation officers to ensure that the first-time testers and repeat testers are adequately tracked. We divided the total number of KPs who tested HIV positive (numerator: first-time testers + previously known HIV–positive testers) by the total number of KP who received HIV testing services between October 1, 2016, and September 30, 2017, to measure the HIV prevalence.

### The 2014 Integrated Biological Behavioral Sentinel Survey

The 2014 IBBSS was conducted by the Federal Ministry of Health through the National AIDS & STIs Control Programme (NASCP) and other stakeholders and used respondent-driven sampling, a modified form of snowball sampling to identify hard to reach populations [32], to select MSM and PWID. The 2014 IBBSS was conducted in 14 states, and only 4 of the 7 states in our study (ie, Lagos, Rivers, Federal Capital Territory, and Cross River) had data on seroprevalence for MSM and PWID recorded in the 2014 IBBSS report. The goal of the IBBSS is to obtain serological, behavioral, and HIV service coverage data on key and vulnerable populations. Members of the communities, nongovernmental organizations working with the target populations, and key informants for each target group (recruited as seeds) helped identify various locations where the target groups could be found. Seeds were identified through the nongovernmental organizations’ networks that historically provide support and services for MSM and PWID. A list of sites where the population groups were located, how and when they can be reached for information and services, and the essential distinguishing characteristics of these sites were prepared and used for the 2014 IBBSS. All eligible key and vulnerable populations were tested for HIV, and results were documented in survey forms.

### Study Population

All MSM and PWID aged ≥15 years with a documented HIV test result were included. At the time of analysis and in line with the Nigeria KP program, HIV testing data were disaggregated into 4 age categories, 15-19, 20-24, 25-49, and 50+ years. The age classification for the HIV test data was based solely on the nature of the KP program. MSM was defined as self-identification as male and report of oral or anal sex with a man in the previous 12 months [35]. A PWID was defined as an individual meeting one of the following conditions: self-reporting ever injecting any illicit drug and having a visible injection site on the body and self-reporting injecting illicit drugs in the past month [36]. All KP groups were screened before enrollment by known MSM or PWID recruited as peer navigators by the national KP program to ensure clients were members of the target population.

### Statistical Analyses

We analyzed aggregate-level data from index testing, mobile, and voluntary counseling and testing modalities by counting the number of KPs who received HIV testing services via the The President's Emergency Plan for AIDS Relief (PEPFAR) and the Nigerian KP program between October 1, 2016, and September 30, 2017, at the LGA level. We georeferenced all MSM and PWID who accessed HIV testing services at the LGA level and performed 4 analyses. First, we constructed maps of HIV seroprevalence among MSM and PWID. Second, we used spatial autocorrelation (global Moran I) statistics to measure the degree to which HIV seroprevalence was clustered, dispersed, or randomly distributed. Under the null hypothesis, the expected value is that there is no pattern of HIV infection in selected LGAs. Moran I values range from –1 (indicating perfect dispersion) to 1 (indicating perfect spatial clustering). Third, we used hots pot analysis in the ArcGIS software (Environmental Systems Research Institute) to calculate the Getis-Ord Gi* statistic for each LGA. The resultant *z* score identified where LGAs with either high or low HIV seroprevalence cluster spatially. Each LGA was analyzed within the context of neighboring LGAs: the larger the *z* score, the more intense the clustering of high seroprevalences or hot spots. Similarly, smaller *z* scores indicated clustering of low seroprevalences. To be a statistically significant hot spot, an LGA required a high HIV seroprevalence and to be surrounded by other LGAs with high HIV seroprevalence. For the 2 study groups, the map scale used varied: 1:25,000 was used for MSM and 1:42,000 for PWID. A resolution of 300 dpi was consistent throughout the 2 maps. Fourth, our study's calculated HIV seroprevalence was compared to the HIV seroprevalence among MSM and PWID from the 2104 IBBSS. Using Stata 14 (StataCorp) we developed 95% CIs of HIV seroprevalence by state with the normal approximation. We created dot plots to compare the distribution of the 2 different HIV seroprevalence results (study vs IBBSS) between MSM and PWID in Lagos, Rivers, the Federal Capital Territory, and Cross River.

### Ethical Reviews

This analysis was conducted with routine data gathered through the national KP program. Informed consent was obtained for all clients who were tested for HIV in line with the HIV testing service policy of Nigeria. Ethical approval was obtained from the Federal Capital Territory, Health Research Ethics Committee, Nigeria (approval no. FHREC/2019/01/122/23-12-19). This study only analyzed anonymized and deidentified data. The 2014 IBBSS received appropriate ethical clearance from the National Health Research and Ethics Committee before the commencement of the survey.

## Results

### Overall HIV Seroprevalence by Age and Sex

Of the 26,423 MSMs and 9474 PWID who received HIV testing between October 1, 2016, and September 30, 2017, a total of 3209 MSM and 1126 PWID tested HIV positive for an overall seroprevalence of 12.1% (95% CI 9.7-13.1) and 11.8% (95% CI 9.312.7), respectively (Table 1). MSM aged 50 years and older had a considerably higher HIV seroprevalence (43/126, 34.1%,) than those aged 15-19 years (190/2648, 7.17%,). Middle-aged PWID (25-49 years old) had the highest HIV seroprevalence (905/6203, 14.58%,) over other PWID age groups. HIV seroprevalence among female PWID was twice as high as that among male PWID (female: 467/2485, 18.79%; male 659/6989, 9.42%). Female PWID aged 25-49 years had the highest HIV seroprevalence (345/1399, 24.66%,), while the seroprevalence among male PWID in the same age group was 11.65% (560/4804; Table 1).

**Table 1:** HIV Seroprevalence among MSM and PWID by age, sex, and study locations in Nigeria, September 30, 2017.

State by age group		No. of individuals tested for HIV				No. of individuals who tested HIV positive				SP^a^ (%) among MSM (n=26423)		SP (%) among male PWID (n=6989)		SP (%) among female PWID (n=2485)
		MSM^b^ (n=26,423)	PWID^c^		MSM (n=3209)		PWID							
			Male (n=6989)	Female (n=2485)			Male (n=659)	Female (n=467)						
**Akwa Ibom State **														
	15-19 y	227	75	29	25		2	3	11.0		2.7		10.3	
	20-24 y	1134	281	82	53		13	20	4.7		4.6		24.4	
	25-49 y	2242	1033	188	490		159	66	21.9		15.4		35.1	
	50+ y	32	13	3	13		4	2	40.6		30.8		66.7	
**Benue State**														
	15-19 y	264	42	14	16		6	3	6.1		14.3		21.4	
	20-24 y	1093	251	66	70		10	10	6.4		4.0		15.2	
	25-49 y	1117	875	117	282		85	40	25.2		9.7		34.2	
	50+ y	4	31	5	4		6	1	100.0		19.4		20.0	
**Cross River State**														
	15-19 y	327	100	64	30		3	11	9.2		3.0		17.2	
	20-24 y	1495	358	176	145		11	27	9.7		3.1		15.3	
	25-49 y	2193	946	344	349		149	122	15.9		15.8		35.5	
	50+ y	31	9	1	11		4	0	35.5		44.4		0.0	
**Federal Capital Territory**														
	15-19 y	753	28	41	21		0	0	2.8		0.0		0.0	
	20-24 y	2401	318	280	154		6	19	6.4		1.9		6.8	
	25-49 y	2332	677	293	243		24	26	10.4		3.5		8.9	
	50+ y	10	2	1	5		1	1	50.0		50.0		100.0	
**Lagos State**														
	15-19 y	496	6	23	34		0	5	6.9		0.0		21.7	
	20-24 y	1375	44	76	167		2	5	12.1		4.5		6.6	
	25-49 y	731	278	219	99		4	26	13.5		1.4		11.9	
	50+ y	10	96	23	2		1	1	20.0		1.0		4.3	
**Nasarawa State**														
	15-19 y	227	51	21	7		1	1	3.1		2.0		4.8	
	20-24 y	1294	369	181	97		8	13	7.5		2.2		7.2	
	25-49 y	1193	668	238	153		68	65	12.8		10.2		27.3	
	50+ y	6	3	0	2		1	0	33.3		33.3		—^d^	
**Rivers State**														
	15-19 y	354	7	0	57		2	0	16.1		28.6		—	
	20-24 y	1929	88	0	268		15	0	13.9		17.0		—	
	25-49 y	3120	327	0	406		71	0	13.0		21.7		—	
	50+ y	33	13	0	6		3	0	18.2		23.1		—	
**Overall**														
	15-19 y	2648	309	192	190		14	23	7.2		4.5		12.0	
	20-24 y	10,721	1709	861	954		65	94	8.9		3.8		10.9	
	25-49 y	12,928	4804	1399	2022		560	345	15.6		11.7		24.7	
	50+ y	126	167	33	43		20	5	34.1		12.0		15.2	
Total		26,423	6989	2485	3209		659	467	12.1		9.4		18.8	

^a^SP: seroprevalence.

^b^MSM: men who have sex with men.

^c^PWID: people who inject drugs.

^d^Data not available.

### HIV Seroprevalence by State: Comparison to Previous Surveys

**Figure 1:**
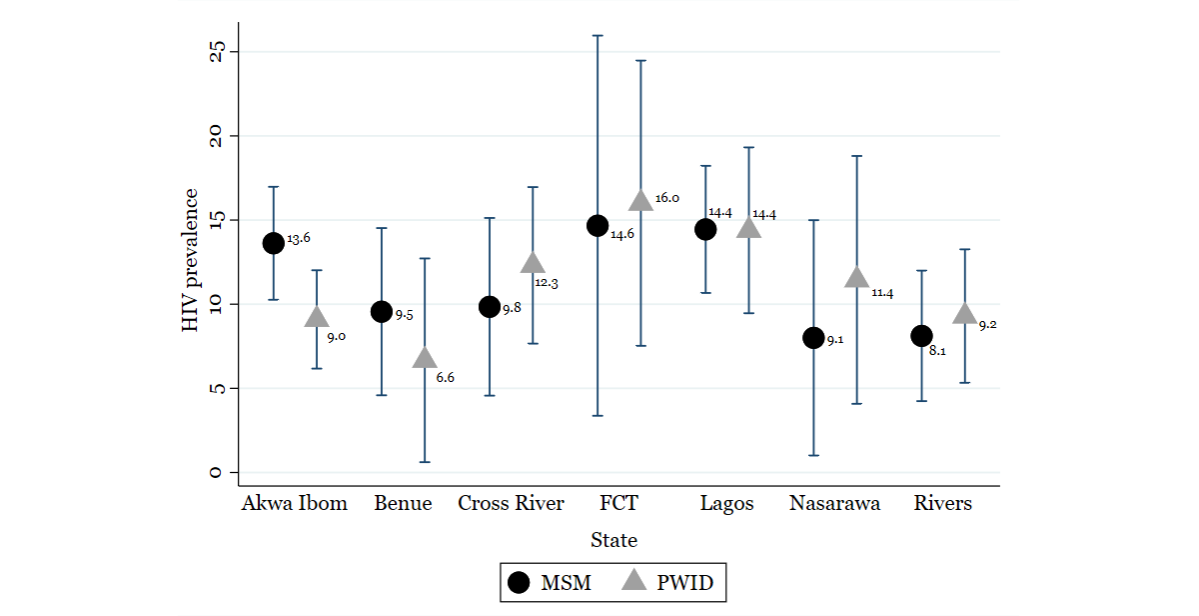
HIV seroprevalence among MSM and PWID by state, September 30, 2017. Mean HIV seroprevalence with 95% CIs. FCT: Federal Capital Territory; MSM: men who have sex with men; PWID: people who inject drugs.

**Figure 2:**
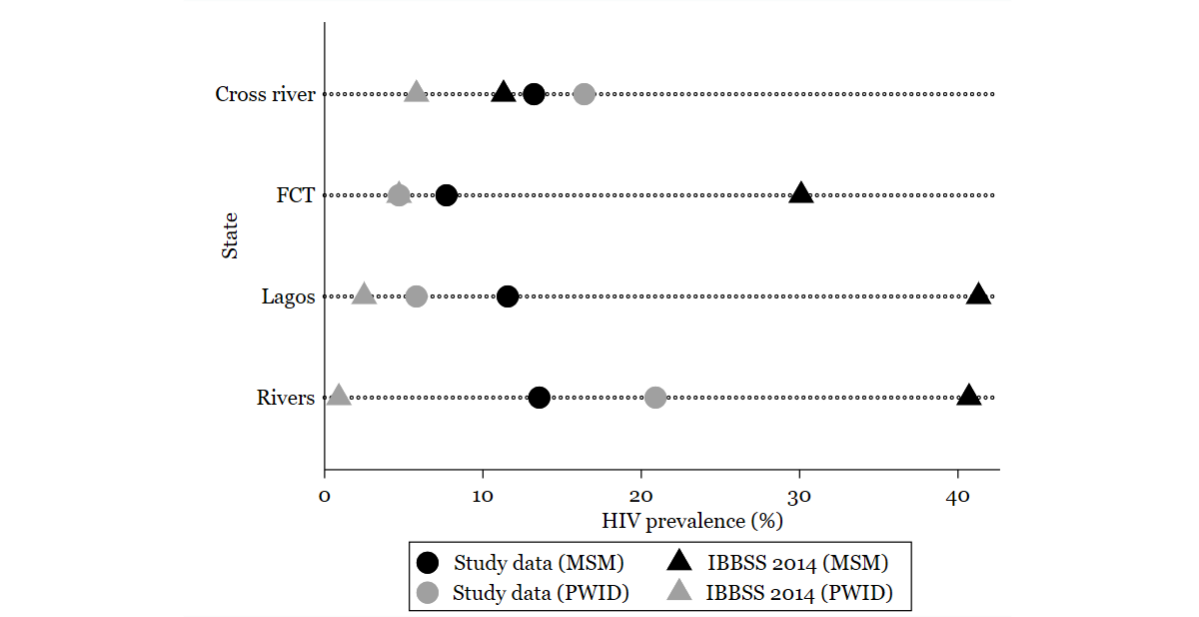
Comparison of HIV seroprevalence from the study to that in the 2014 IBBSS. The black circles and black triangles represent HIV seroprevalence among MSM as seen in our analysis and the 2014 IBBSS, respectively. The grey circles and grey triangles represent the PWID HIV seroprevalence as seen in our study and the 2014 IBBSS, respectively. FCT: Federal Capital Territory; IBSS: integrated biological and behavioural surveillance survey: MSM: men who have sex with men; PWID: people who inject drugs.

Figure 1 shows the mean HIV seroprevalence for the various states. Among MSM in all states, the mean HIV seroprevalence in the Federal Capital Territory was the highest (14.6%; 95% CI 3.4-25.9), followed by Lagos (14.4%; 95% CI 10.6-18.2), and Akwa Ibom state (13.6%; 95% CI 10.2-16.9). Mean HIV seroprevalence was lowest in Nasarawa (9.1%; 95% CI 1.0-14.9). Similarly, among PWID, mean HIV seroprevalence in the Federal Capital Territory was the highest (16.0%; 95% CI 7.5-24.5), followed by Lagos (14.4%; 95% CI 9.5-19.3). The mean seroprevalence in Cross River was 12.3% (95% CI 7.7-16.9), while that among PWID was the lowest in Benue (6.6%; 95% CI 0.6-12.7; Figure 1). Figure 2 shows HIV seroprevalence for the various states compared to previous estimates from the 2014 IBBSS. Findings from the 2014 IBBSS have demonstrated that HIV seroprevalence increased among MSM: from 13.5% in 2007 to 17.2% in 2010 to 23.0% in 2014. Simultaneously, the seroprevalence among PWID decreased from 5.6% in 2007 to 4.2% in 2010 to 3.4% in 2014.

### Variability of HIV Seroprevalence Within Different States

**Figure 3:**
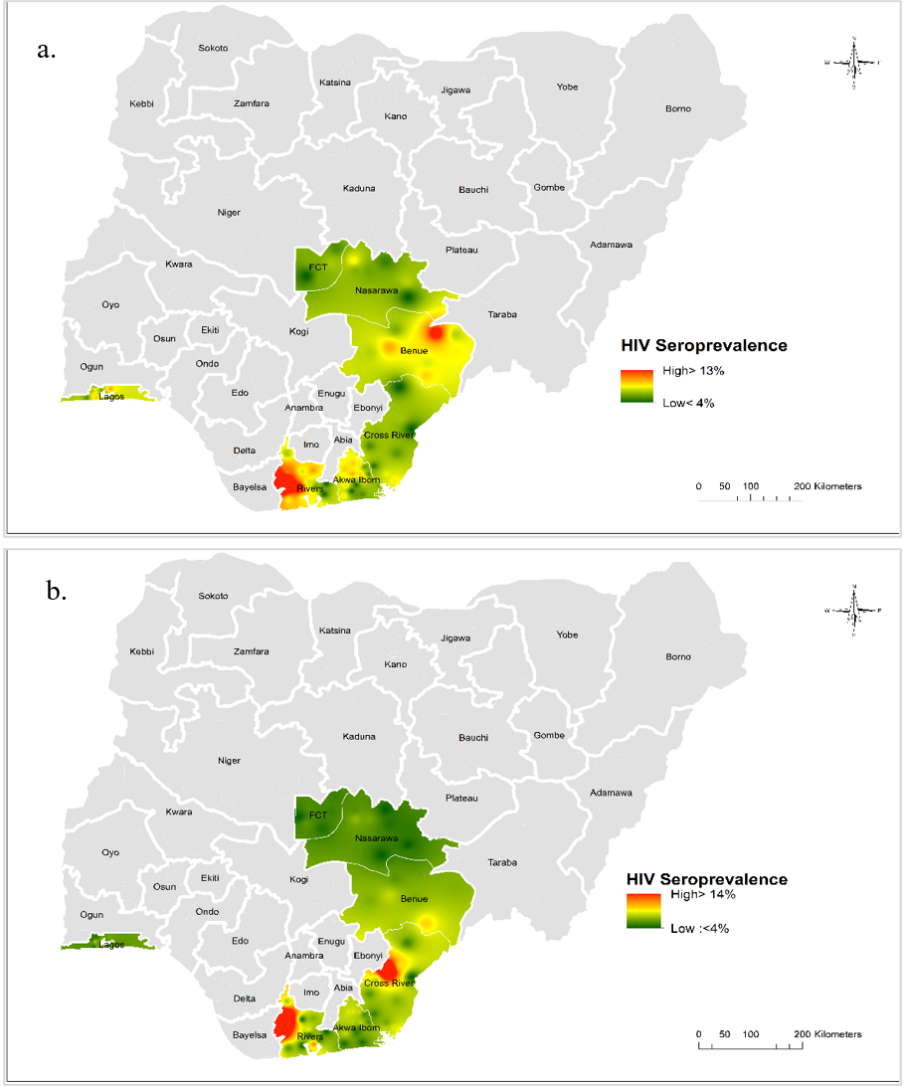
Map of Nigeria showing the interpolated areas with high and low HIV seroprevalence on from October 1, 2016, to September 30, 2017, among men who have sex with men (a) and people who inject drugs (b). Seroprevalence >13% is shown in red, and seroprevalence <4% is shown in green.

**Figure 4:**
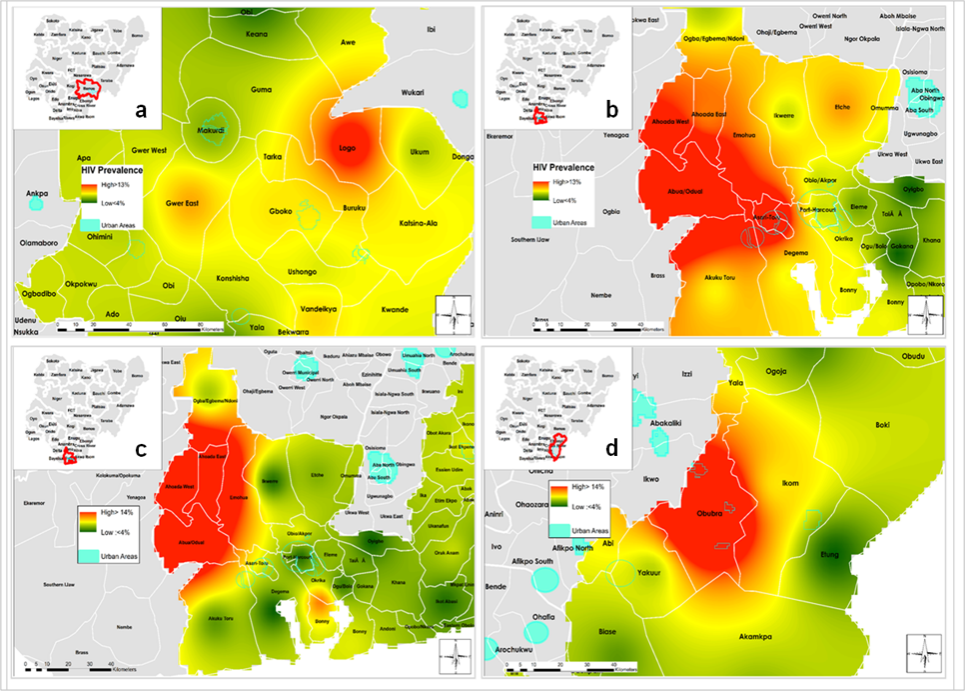
State level map of Nigeria showing urban locations and interpolated areas with high and low HIV seroprevalence by local government area among men who have sex with men (a: Benue; b: Rivers), and people who inject drugs (c: Rivers; d: Cross River). Seroprevalence >13% is shown in red, and seroprevalence <4% is shown in green.

The interpolated HIV seroprevalence map reveals the geospatial distribution of HIV seroprevalence among MSM (Figure 3a, top panel) and PWID (Figure 3b, bottom panel) within various states. Seroprevalence in MSM was higher than in PWID. Large-scale spatial patterns are apparent, and they are more distinct for MSM than for PWID. Among MSM in Benue state, a high seroprevalence (>13%) was evident in the Logo community, while in the Gwer East and Vandeikya LGAs, the seroprevalence was below 10% (Figure 4a). HIV seroprevalence among MSM in Rivers state was the highest in the Asari-Toru, Emohua, Ahoada East, Ahoada West, and Abua/Odual LGAs (Figure 4b). Similarly, HIV seroprevalence was relatively higher in Rivers among PWID who live outside the mapped urban area settlements in Ahoada East, Ahoada West, Emohua, Abua/Odual, and Bonny (Figure 4c). Notably, in Rivers state, high HIV seroprevalence among MSM was recorded in large urban area settlements in the Asari-Toru LGA. Seroprevalence in Cross River among PWID was highest in urban area settlements in the northern region of the Obubra LGA (Figure 4d).

### Comparison of Program Data and the IBBSS 2014

Clear state differences in HIV seroprevalence between the 2014 IBBSS and our program data were observed for MSM in Lagos (41.3% vs 11.6%), Rivers (40.7% vs 13.6%), and the Federal Capital Territory (30.1% vs 7.7%). Seroprevalence from program data was higher than the seroprevalence from the 2014 IBBSS for MSM in the Cross River state (13.2% versus 11.3%). Seroprevalence among PWID was higher in our program data compared to the 2014 IBBSS data in Lagos (5.8% vs 2.5%], Rivers (20.9% vs 0.9%), and Cross River (16.4% vs 5.8%).

### Detection of Spatial Patterns and Hot Spots of HIV Seroprevalence

The global spatial autocorrelation Moran I statistics confirmed the clustered distribution of HIV seroprevalence among MSM and PWID. We found more significant clustering among PWID (*z* score=4.03; *P*<.001) compared to MSM (*z* score=2.29; *P*<.02). Getis-Ord-Gi* statistics indicated significant clusters of HIV infection among MSM and PWID (Multimedia Appendix 1) that were confined to LGAs in the North Central (Abuja Municipal Area Council, Bwari, Karu, Keffi, and Gwer East) and South South (Calabar Municipal, Calabar South, Degema, Odukpani, and Akpabuyo) regions of the country.

## Discussion

### Principal Results

Our study shows a substantial geographic variation in the HIV seroprevalence of both MSM and PWID throughout the study areas. Similarly, mounting evidence suggests a large geographical variation in the sub-Saharan Africa HIV epidemic [37,38]. Although data on MSM in Eastern and Southern Africa are limited, HIV seroprevalence ranges from 2% in Angola to 31% in Zimbabwe [39]. The UNAIDS 2019 report suggests condom use by MSM exceeded 70% in South Africa, Kenya, and Rwanda, and was above 50% in Angola, Comoros, Eswatini, Madagascar, and Mauritius. Lesotho, Malawi, and Tanzania reported levels below 50% at 46%, 44%, and 14%, respectively [39]. Overall, around 1 in 5 (20%) MSM in the region are estimated to be living with HIV [36]. Evidence suggests the majority of the region’s MSM engage in heterosexual sex, often with wives or other long-term female partners [39]. The HIV epidemic among MSM is therefore interlaced with the epidemic in the wider population [40]. Kenya, Madagascar, Mauritius, Mozambique, South Africa, Tanzania, and Uganda are all home to PWID populations. Overall, just under a third (30%) of people who inject drugs in these regions are believed to have HIV, and the proportion of new HIV infections arising from injection drug users is estimated to be as high as 75% to 80% percent in Eastern European countries and some areas of South and Southeast Asia [41]. According to previous study, the majority of PWID in Kenya (89%) reported injecting in groups, and nearly a quarter (27.6%) of those who reported distributive syringe sharing (sharing used syringes with others) reported being infected with HIV [41]. In Tanzania, it is estimated that 15.5% of PWID are living with HIV, with HIV prevalence among women who inject drugs thought to be higher than among their male counterparts [42]. Between 85% to 96% of female PWID engage in transactional sex, and half of those working in transactional sex reported they had never used a condom in the previous 30 days, although just 23% said they still used condoms [43]. In 2018, an estimated 21.8% of people who inject drugs in South Africa lived with HIV [10]. A 2015 study in 5 South African cities found 32% of men and 26% of women who inject drugs regularly shared syringes and other injecting equipment, and nearly half reused needles [44]. Evidence from Kenya, Mauritius, Seychelles, and Tanzania suggests many PWID acquire HIV before the age of 25 years [26]. The interpolated maps in our analysis identified significant clustered patterns of the spatial distribution of HIV seroprevalence in Nigeria that would have been missed using macro-level national data. Spatial clustering of HIV seroprevalence, as shown in our study, can be due to geographic mobility trends, high rates of risky injection and sexual behaviors, and stigma and discrimination. Studies have consistently linked residential instability with high-risk behaviors (eg, syringe sharing and exchanging sex for drugs or money) and HIV infection [45]. Overall, HIV seroprevalence in our study ranged from 9.6% to 13.1% among MSM and from 9.3% to 12.7% among PWID. Seroprevalence was disproportionately distributed across the states. The median seroprevalence of 20.0% (95% CI 1.0-24.0) and 19.0% (95% CI 2.2-23.0) for MSM and PWID, respectively, in the Federal Capital Territory, and 18.5% (95% CI 3.0-23.0) for PWID in Lagos shows a concentrated epidemic among these KPs in Nigeria. Surveillance data have shown that women carry the highest burden of HIV on the African continent, with national-level statistics reporting that women have a higher HIV seroprevalence and incidence than men. These reports are substantiated by findings from our study, which showed that HIV seroprevalence among female PWID was twice as high as that in male PWID (female: 467/2485, 18.79%; male 659/6989, 9.42%).

Our study shows that, relative to the other age groups, MSM aged 50 years and older have higher HIV seroprevalence in all study areas. Our observation is consistent with previous studies suggesting a steady increase in the number of people living with HIV who are 50 years of age and older [46]. A study from Zimbabwe found that more than half of the adults aged 50 years and older included in the analysis had seroconverted after their 50th birthday [47]. Household survey results indicate that people 50 years of age and above are less likely than younger people 15-49 years old to have ever been tested for HIV [42]. In comparison, older persons were found to be less likely to use condoms during the most recent intercourse [48]. In our study, HIV seroprevalence among female PWID was twice as high as that in male PWID, with female PWID having higher HIV seroprevalence across all age groups. Data disaggregated by sex from PWID show considerable geographic variation in HIV prevalence among women who inject drugs (seroprevalence range 0%-65%) [49]. In Tanzania, 55% of young women (aged 17-25 years) who injected drugs were HIV positive compared with 12% of young men who injected drugs [50]. In a review of sex differences in settings in which HIV seroprevalence among PWID is greater than 20%, women who inject drugs were more likely than their male counterparts to be living with HIV infection (pooled odds ratio 1.18; 95% CI 1.10-1.26) [51]. These findings indicate that the considerable heterogeneity in HIV seroprevalence between and within the study states may be clarified by sex and age-specific behaviors or characteristics.

Among MSM, the substantial decrease in HIV seroprevalence observed in our study data compared to the IBBSS 2014 could be attributed to better surveillance [52] and effectiveness of HIV interventions in the study states over 4 years. The minimum comprehensive package of HIV interventions implemented in these states and targeted at the general population and key population groups included community and facility testing for HIV, linkage of HIV-positive individuals to antiretroviral treatment, and HIV treatment retention to improve viral suppression. The findings from our study are substantiated by reports from the NAIIS 2018, which showed a national HIV seroprevalence in Nigeria of 1.4% among adults aged 15-49 years compared to previous national seroprevalence estimates of 3.4% from the 2012 National HIV & AIDS and Reproductive Health Survey. The NAIIS also showed that Nigeria had demonstrated steady progress on increasing access to treatment for people living with HIV, adopting a test and treat policy in 2016. Among PWID, our results indicated a higher seroprevalence compared to the 2014 IBBSS. The disproportionately high seroprevalence among PWID in our study compared to the 2014 IBBSS could be linked to the HIV infection distribution pattern among PWID that suggests overlapping risk groups with multiple transmission routes. For example, some PWID are sex workers, buy or trade drugs for sex, or are MSM, and may acquire HIV through sexual and injecting routes [53]. The differences between the program data and the IBBSS in HIV seroprevalence estimates may be due to differences in the sample size and HIV testing strategies. The differences in the proportion of the KPs tested for HIV by the state in the IBBSS suggests low participation in the survey mainly attributable to fear of stigma and criminalization by the Same-Sex Marriage Prohibition Act and discrimination against KPs. Although the IBBSS is implemented by the Federal Ministry of Health through NASCP, the national KP program is implemented by nongovernmental organizations through technical, logistics, and funding support from PEPFAR and bilateral development agencies (ie, United States Agency for International Development [USAID], Centers for Disease Control and Prevention, and the Department of Defense). The program provides all KP types with differentiated HIV testing and treatment services in a friendly and safe space at the community level. KP are extremely mobile [54], but they are more likely to participate in the program because of their regular involvement and consistent engagement with program personnel, i.e., program staff who are KP, and are hired as peer-outreach staff and HIV assessment counselors.

Recent studies outside sub-Saharan Africa have established a high seroprevalence of HIV among overlapping high-risk populations such as PWID, MSM, and female sex workers. There are multiple transmission routes. Epidemiological studies from 2003 to 2015 in Tijuana, Mexico, reported a pooled HIV seroprevalence of 3.4% among male PWID, but 8% among male PWID who have sex with men, and 5% among male PWID who are clients of female sex workers [51]. Similarly, pooled studies from 2003 to 2015 reported a 5% HIV seroprevalence among female sex workers, indicating a rise from 6.7% among female PWID and 7.3% among female sex workers who inject drugs [51]. The 2018 National Survey on Drug Use and Health in Nigeria reported that PWID constitutes a sizeable proportion of high-risk drug users in Nigeria, with 1 in 5 high-risk drug users injecting drugs [55]. The most common drugs injected in the past year were pharmaceutical opioids, followed by cocaine and heroin. Women who injected drugs were more likely than men to engage in high-risk sexual behaviors, further compounding their risk for acquiring HIV, among other infections [55]. These results corroborate findings from our study that showed a higher seroprevalence of HIV among female PWID than among male PWID.

### Limitations

A fundamental limitation of our study was that criminalization through the Same-Sex Marriage Prohibition Act and discrimination against KPs has driven several KPs underground, limiting the study's overall representation. Consequently, continued discrimination and criminalization of KPs impede access to health services and willingness to participate in surveys. In 2014, the Nigerian government increased the punishment for homosexuality to 14 years in jail. Anyone who assists homosexual couples may face up to 10 years in prison [56]. Mass arrests of suspected gay men in Nigeria have followed. For example, in July 2017, the police arrested 40 men at a private house party [57]. Laws criminalizing homosexuality such as these have pushed MSM into hiding, making them more vulnerable to HIV [58].

At the time of analysis, and in line with the Nigeria KP program, HIV testing data was disaggregated into 4 age categories, 15-19, 20-24, 25-49, and 50+ years. The age range for HIV test results was based purely on the nature of the program, and thus the age range of 25-49 years could not be broken down into broader age categories, which might have influenced the variability of HIV seroprevalence in that age group between MSM and PWID. Given the length of the HIV testing data period (between October 2016 and September 2017), the analysis findings provide valid benchmark evidence for potential program reviews evaluating improvements in the adoption of HIV testing services and tracking practices relevant to the study of HIV seroprevalence variability among KPs. Program data used for this analysis were collected at an aggregate level and did not include individual or patient-level socioeconomic and clinical characteristics of the study population that might have provided further insights into the variation of HIV seropositivity. Due to the unavailability of individual-level data, spatial regression analysis could not be conducted.

Consequently, the unavailability of the individual-level variables might have influenced the spatial analysis results, but the study identified significant subnational clusters of HIV infection that can be prioritized for tailored HIV prevention and treatment interventions. Identifying areas where the burden of HIV infection is concentrated might play a key role in identifying populations at higher risk of infection. Despite our study method of recruiting MSM and PWID for HIV testing being the same as that for the 2014 IBBSS, sample size variations might have influenced findings. Our study analyzed 89% of MSM and 84% of PWID compared with the 2014 IBBSS.

### Future Work

Further studies are recommended to determine the immediate effect of this prohibitive act on stigma, discrimination, and engagement among MSM in HIV prevention and treatment services in Nigeria. We suggest more studies to explore the risk factors associated with spatial clustering of HIV infection between MSM and PWID.

### Optimizing the Impact of the KP Program

The large sample of KP data collected and used in our study compared to other previous studies can be attributed to the peer outreach model. In this model, KPs are trained as peer-outreach workers to increase demand for tailored HIV services, improve the quality of behavior change communication, and increase access to HIV testing services—the starting point of the KP cascade—via social networks. This approach yielded veritable insights on the KP groups at the LGA level and offers a unique opportunity to estimate national and subnational key population size by scaling up the peer outreach method for data collection in HIV programming. As reflected in our study, the large sample of MSM and PWID data indicates that KPs can be effectively mobilized for HIV testing and treatment despite the legal, policy, and social barriers. Based on the 2018 national KP size estimation carried out by NASCP [59], the total KP in Nigeria is estimated to be 720,000. About three-quarters (69%) of the KP have been tested for HIV under the KP program. In our program, KP’s high coverage in HIV testing offers an additional viewpoint on the access and uptake of HIV testing services at the community levels. We acknowledge that while a significant proportion of KPs living with HIV have been tested for HIV by the national program, the study sample might not be representative, as our study did not compare KP characteristics included or not included in the national program. In our study, this was another limitation.

Two studies in Uganda confirmed that program data routinely collected from the prevention of mother‐to‐child transmission programs can be used to monitor HIV prevalence trends [60,61]. Nigeria has a mixed epidemic, and HIV prevalence is disproportionality distributed among KPs. As we must wait for every 5-years to conduct an IBBSS, which depends entirely on the availability of funds, it is imperative to ensure frequent measurements to monitor changes in new HIV infections. The use of routine program data from groups with high-risk behaviors like MSM and PWID can help monitor trends in HIV seroprevalence at the community level. Identification of high HIV prevalence regions using routine program data can guide timely program management decisions on potential locations to scale up antiretroviral treatment interventions (the era of test and treat). Across several sub-Saharan Africa countries (including Nigeria), UNAIDS currently uses routine program data in Spectrum Software to estimate the national HIV prevalence and people living with HIV burden. The Spectrum package uses the HIV prevalence over time from routine program data or survey data together with demographic information and epidemiological assumptions to model age-specific HIV prevalence, incidence and mortality rates, and the total number of people living with HIV [62].

### Conclusions

To our knowledge, this study represents the most extensive investigation on HIV seroprevalence among KPs in Sub-Saharan Africa. We showed that there is significant clustering and subnational variation in HIV seroprevalence among MSM and PWID. The geographical variations in HIV seroprevalence revealed by our analysis mean that HIV infection is disproportionately spread across Nigeria and confined to a particular region. To inform HIV prevention and care strategies in the more generalized HIV epidemics across sub-Saharan Africa, including in Nigeria, epidemiological assessment of the geographical heterogeneity of HIV among MSM and PWID is essential. Our results suggest that HIV seroprevalence among middle-aged adult female PWID is disproportionately greater than among male PWID. PWID could transmit their HIV infection to their sexual contacts and injecting partners, which in turn may lead to the spread of HIV to the general population, particularly in the context of transactional sex. Interventions that significantly focus on women and their sexual and injecting partners are essential to address these high HIV acquisitions and transmission risks among KPs, particularly PWID. The substantial decrease in HIV seroprevalence among MSM as observed in our study data compared to the 2014 IBBSS suggests improvements in the national HIV prevention and treatment programs over the past 4 years. Understanding heterogeneity in mixing patterns among KPs in concentrated HIV epidemics may help in designing more effective interventions. We recommend the use of routine HIV testing service program data for the implementation of HIV infection surveillance among KPs. They should serve as an essential input for statistical models that estimate the national and subnational burden and incidence of HIV using estimation and projections tools.

## Appendix

Multimedia Appendix 1 Hot spot analysis (Getis-Ord-Gi*) of HIV infection distribution among men who have sex with men (a. left) and people who inject drugs (b. right).

## Abbreviations

IBBSS: integrated biological and behavioral surveillance survey
KP: key population
LGA: local government area
MSM: men who have sex with men
NAIIS: Nigeria AIDS Indicator and Impact Survey
NASCP: National AIDS & STIs Control Programme
PEPFAR: The President's Emergency Plan for AIDS Relief
PWID: people who inject drugs
UNAIDS: the Joint United Nations Programme on HIV/AIDS
USAID: United States Agency for International Development
