# Supplementary material for: Geographical Disparities in HIV Seroprevalence Among Men Who Have Sex with Men and People Who Inject Drugs in Nigeria: Exploratory Spatial Data Analysis
Source: JMIR Public Health Surveill. 2021 May 24;7(5):e19587. doi: 10.2196/19587 (PMC8185612; doi:10.2196/19587)
Supplement: Multimedia Appendix 1 [file publichealth_v7i5e19587_app1.pdf]

**Annex 1**

**Table 2: Hot spot analysis (Getis-Ord-Gi*) of HIV infection distribution among MSM (a. left) and PWID (b. right)**

Only LGAs with a p value < 0.05 are shown.

| A | | | | | B | | | | |
|---|---|---|---|---|---|---|---|---|---|
| LGA | MSM (# HIV Positive) | GiZScore | GiPvalue | GiBIN | LGA | PWID (# HIV Positive) | GiZScore | GiPvalue | GiBIN |
| | | | | | | | | | |
| Calabar Municipal | 234 | 1.977624 | 0.047971 | 2 | Gwer East | 71 | 1.897651 | 0.057742 | 1 |
| AMAC | 430 | 3.282569 | 0.001029 | 3 | Calabar South | 97 | 4.007088 | 0.000061 | 3 |
| Bwari | 69 | 4.205538 | 0.000026 | 3 | Calabar Municipal | 157 | 4.78932 | 0.000002 | 3 |
| Karu | 155 | 3.559387 | 0.000372 | 3 | Obubra | 3 | 2.511684 | 0.012016 | 2 |
| Degema | 58 | 2.157567 | 0.030962 | 2 | Keffi | 104 | 2.046563 | 0.040701 | 2 |
| Port-Harcourt | 204 | 2.645243 | 0.008163 | 3 | Bakassi | 100 | 2.92129 | 0.003486 | 3 |
| | | | | | Akpabuyo | 94 | 4.990284 | 0.000001 | 3 |
